# Integration and Differentiation of Transplanted Human iPSC-Derived Retinal Ganglion Cell Precursors in Murine Retinas

**DOI:** 10.3390/ijms252312947

**Published:** 2024-12-02

**Authors:** Qiannan Lei, Rong Zhang, Fa Yuan, Mengqing Xiang

**Affiliations:** 1 State Key Laboratory of Ophthalmology, Guangdong Provincial Key Laboratory of Ophthalmology and Visual Science, Zhongshan Ophthalmic Center, Sun Yat-sen University, Guangzhou 510060, China; leiqiannan@gzzoc.com (Q.L.); zhangr253@mail2.sysu.edu.cn (R.Z.); yuanf28@mail2.sysu.edu.cn (F.Y.); 2 Guangdong Provincial Key Laboratory of Brain Function and Disease, Zhongshan School of Medicine, Sun Yat-sen University, Guangzhou 510080, China

**Keywords:** retinal organoid, retinal ganglion cell, human-induced pluripotent stem cells, cell transplantation, BRN3B

## Abstract

Optic neuropathy such as glaucoma, stemming from retinal ganglion cell (RGC) degeneration, is a leading cause of visual impairment. Given the substantial loss of RGCs preceding clinical detection of visual impairment, cell replacement therapy emerges as a compelling treatment strategy. Human-induced pluripotent stem cells (hiPSCs) serve as invaluable tools for exploring the developmental processes and pathological mechanisms associated with human RGCs. Utilizing a 3D stepwise differentiation protocol for retinal organoids, we successfully differentiated RGC precursors from hiPSCs harboring a BRN3B-GFP RGC reporter, verified by GFP expression. Intravitreal transplantation of enriched RGC precursors into healthy or N-methyl-D-aspartate (NMDA)-injured mice demonstrated their survival, migration, and integration into the proper retinal layer, the ganglion cell layer, after 3 weeks. Notably, these transplanted cells differentiated into marker-positive RGCs and extended neurites. Moreover, enhanced cell survival was observed with immunosuppressive and anti-inflammatory treatments of the host prior to transplantation. These data underscore the potential of transplanted RGC precursors as a promising therapeutic avenue for treating degenerative retinal diseases resulting from RGC dysfunction.

## 1. Introduction

The human retina, a component of the central nervous system (CNS), is a highly complex tissue that lines the inner surface of the back of the eyeball. Retinal ganglion cells (RGCs), as the sole output neurons of the retina, play an important role in visual function and transmit visual information from the retina to the brain structure that specializes in processing visual information through the optic nerve and optic tract. RGC injury is a common feature of many pathologies that cause vision loss, including optic neuropathies such as Leber hereditary optic neuropathy (LHON), dominant optic atrophy, and glaucoma, which is the second leading cause of blindness worldwide [1,2]. Elevated intraocular pressure is considered a significant risk factor for glaucoma. Most treatment strategies, such as eye drops and laser surgery, aim to reduce intraocular pressure. However, these strategies cannot prevent further degeneration of RGCs and are ineffective for treating optic nerve damage caused by non-ocular pressure-related glaucoma [3].

As part of the postmitotic CNS, retinal neurons such as RGCs are usually unable to regenerate themselves, and at present, the ideal treatment for degenerative retinal diseases remains elusive. Therefore, there is an urgent need to develop alternative treatment strategies for degenerative retinal diseases such as glaucoma. Unlike traditional medical treatments, a better way to reconstruct vision in glaucoma patients may be to replace RGCs by autologous transplantation or xenotransplantation. Although the supply of RGCs from original donors is limited, stem cells derived from embryos and adult and somatic cells have been shown to be able to differentiate into retinal progenitor cells for potential use in cell therapy of glaucoma [4,5]. In addition, RGCs and RGC-like cells have been derived from stem and somatic cells, including embryonic stem cells (ESCs) [6,7,8,9,10,11], induced pluripotent stem cells (iPSCs) [7,12], Müller cells [13,14], and fibroblasts [15,16,17].

ESCs are a promising choice for the potential treatment of degenerative retinal diseases, but ethical issues hinder their clinical application in humans. iPSCs are less affected by ethical issues and host immune system response after transplantation. In fact, a large number of somatic cells have been reprogrammed into iPSCs, including fibroblasts, keratinocytes, melanocytes, adipose cells, peripheral blood cells, periosteum membrane cells, hepatocytes, and amniocytes [18]. Urine cells (UCs) provide us with a practical and unlimited source of human cells for reprogramming, and this non-invasive way of obtaining human cells would largely increase the patients’ compliance. In the past, we have optimized a non-integrative method of reprogramming urine cells into iPSCs [19].

In this study, we utilized a BRN3B-GFP human iPSC (hiPSC) line previously generated by CRISPR/Cas9 gene editing [20], then induced retinal organoids from these hiPSCs by 3D instead of 2D culture because these organoids contain all major retinal cell types and display a distinct layering that closely mimics the in vivo retinal morphology. Building on the work of Sasai et al., who pioneered the creation of self-organizing optic cups using 3D culture [21,22], we were able to obtain a high-quality and abundant supply of RGC precursors suitable for transplantation. The developmental stage of grafted cells has been shown to be crucial for functional replacement of photoreceptors, with photoreceptor precursor cells most effective [23], suggesting a possible stage dependence for transplanted RGCs as well. In this work, conducted on healthy mice and a model of RGC depletion induced by NMDA, we also considered the microenvironment of transplanted cells, characterized by microglia accumulation and chondroitin sulfate proteoglycan deposition, which hinder cell integration [14]. A previous study has demonstrated that anti-inflammatory therapy and local extracellular matrix degradation can improve cell survival and migration [14]. With these in mind, we developed a protocol for the differentiation of BRN3B-GFP hiPSCs into retinal organoids, enrichment of RGC precursors, and their transplantation onto the inner retinal surface. We assessed the outcome through immunohistochemical analysis of the transplanted retina and observed proper integration and differentiation of grafted human RGC precursors within the murine host retina.

This study serves as a "proof of concept” for retinal RGC transplantation therapy, demonstrating its potential as a viable treatment option. Combining the transplantation of enriched RGC precursors with anti-inflammatory treatment improved the survival of transplanted cells. Our findings contribute to the development of more effective and enduring therapies for patients in advanced stages of retinal degeneration.

## 2. Results

### 2.1. Differentiation of 3D Retinal Organoids from Wild-Type (WT) hiPSCs and BRN3B-GFP hiPSCs

**Figure 1:**
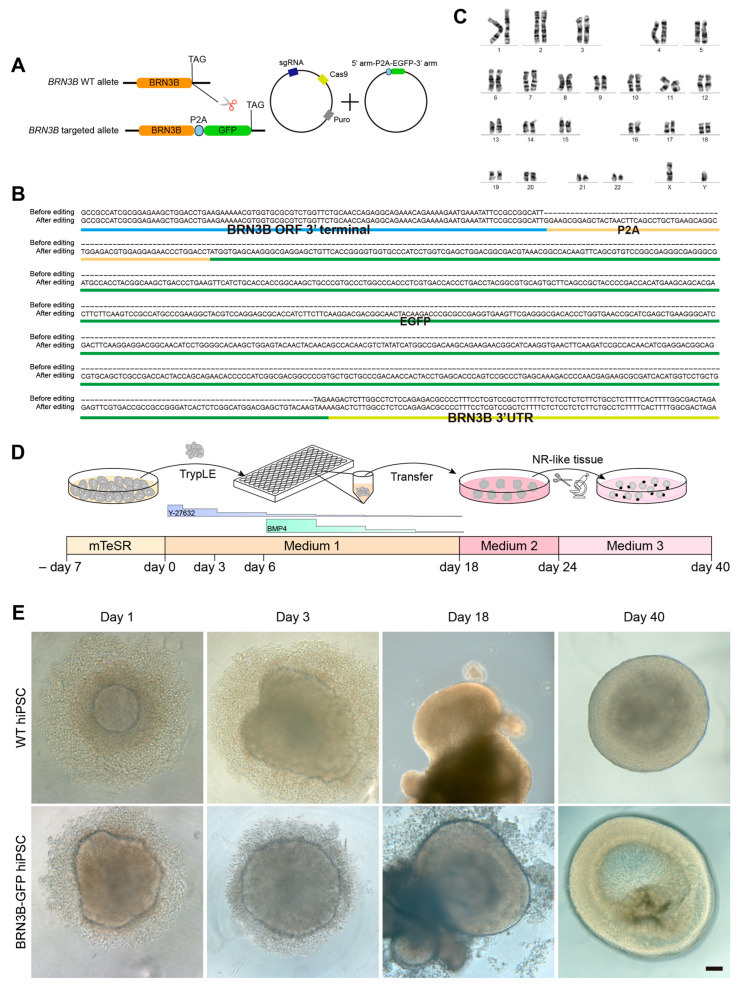
Induction of retinal organoids from BRN3B-GFP hiPSCs. (**A**) Schematic illustration of the insertion of P2A-EGFP into the *BRN3B* locus by CRISPR/Cas9 gene editing. (**B**) Confirmed DNA sequences around the insertion site of the *BRN3B* locus before and after gene editing. (**C**) G-band analysis of BRN3B-GFP iPSCs demonstrated a normal karyotype. The numbers of 1–22 and X, Y represent chromosome numbers. (**D**) Schematic of the retinal organoid differentiation procedure. (**E**) Microscopic images of the cell aggregates and retinal organoids derived from both WT and BRN3B-GFP hiPSCs. Optic cup-like neuroepithelia appeared by day 18 of induction, which gradually transitioned to layered retinal organoids by day 40. Scale bar: (**E**) 100 μm.

The POU domain transcription factor BRN3B has been demonstrated to serve as a pivotal regulator in the specification and differentiation of RGCs [24,25,26,27,28]. It is indispensable for the development of approximately 70% of mouse RGCs and exhibits specific expression within RGC precursors and mature RGCs in embryonic and postnatal mouse retinas [25,26,27]. Consequently, BRN3B stands out as a specific marker for both early and mature RGCs. We utilized a hiPSC reporter line previously generated by CRISPR/Cas9 gene editing to efficiently label and enrich RGCs from 3D retinal organoids. This technique entailed the fusion of the *eGFP* reporter gene to the *BRN3B* open reading frame through a P2A self-cleaving peptide sequence (Figure 1A), leading to the establishment of the BRN3B-GFP hiPSC line [20]. Sequencing confirmed precise insertion at the correct position (Figure 1B). Additionally, the BRN3B-GFP hiPSC line maintained a normal karyotype (Figure 1C).

We then induced BRN3B-GFP hiPSCs into 3D retinal organoids following a published protocol (Figure 1D) [29]. Overall, the differentiation process of the BRN3B-GFP hiPSC line closely resembled that of WT iPSCs. On the first day of hiPSC seeding, spontaneous aggregate formation occurred, gradually progressing into embryoid bodies. And by day 18, enlarged aggregates displayed numerous hemispherical epithelial vesicles. The culture continued until day 24, when we manually dissected neural retina (NR)-like tissues into several fragments using ophthalmic forceps, followed by their continued cultivation. By day 40, distinct layered optic cup/retinal organoids were evident for both WT hiPSCs and BRN3B-GFP hiPSCs (Figure 1E). Thus, similar to WT hiPSCs, the edited BRN3B-GFP hiPSCs are able to differentiate into 3D retinal organoids under proper induction conditions.

### 2.2. Enrichment of Human RGC Precursors from Retinal Organoids for Transplantation

**Figure 2:**
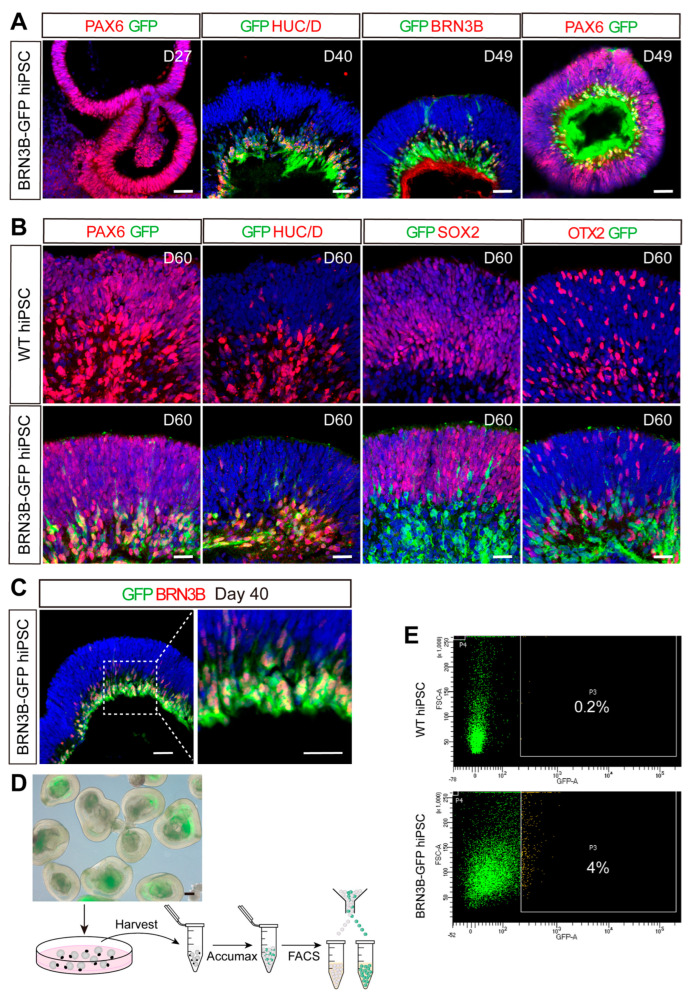
Labeling of RGC precursors by GFP in BRN3B-GFP retinal organoids and their enrichment by fluorescence-activated cell sorting (FACS). (**A**) BRN3B-GFP retinal organoid sections from the indicated time points were double-immunostained for GFP and PAX6, HUC/D, or BRN3B, followed by DAPI counterstaining. GFP^+^ RGC precursors were observed within the inner layer of the organoid after day 40, which co-expressed RGC protein markers HUC/D, BRN3B or PAX6. There was no GFP expression on day 27. (**B**) Sections from 60-day retinal organoids derived from wild-type (WT) and BRN3B-GFP hiPSCs were double-immunolabeled with an anti-GFP antibody and antibodies against the indicated protein markers and then counterstained with DAPI. In BRN3B-GFP retinal organoids, GFP was seen in differentiating RGCs co-expressing PAX6 or HUC/D but not in retinal progenitor cells expressing PAX6 or SOX2 or in photoreceptor precursor cells expressing OTX2. (**C**) Sections of 40-day BRN3B-GFP retinal organoids were double-immunostained with antibodies against GFP and BRN3B and counterstained with DAPI. The right panel is a magnification of the part in the dotted box. RGC precursors (GFP^+^BRN3B^+^) were seen in the inner layer. (**D**) Schematic illustrating the process of enriching GFP^+^ precursors derived from 40-day BRN3B-GFP retinal organoids by FACS. The green dots represent GFP^+^ cells. The image above depicts the morphology of retinal organoids with GFP fluorescence confined to the inner regions. (**E**) Cells expressing BRN3B-GFP were sorted by FACS, resulting in an enrichment of GFP-positive RGC precursors. Single cells from 40-day WT retinal organoids were utilized for control gating (upper panel). Scale bar: (**A**,**C**) 40 μm; (**B**) 20 μm; (**D**) 200 μm.

We performed immunostaining of retinal organoid sections to assess the specificity of GFP reporter gene expression in RGCs derived from the BRN3B-GFP hiPSCs. At day 27 of induction, retinal progenitor cells were strongly labeled for PAX6 but were negative for GFP (Figure 2A), consistent with the lack of BRN3B expression in progenitors. By day 40–49, GFP immunofluorescence emerged within the inner layer of the organoid, colocalizing with RGC markers HUC/D, BRN3B, or PAX6 in the ganglion cell layer, confirming the identity of GFP^+^ cells as RGC precursors and differentiating RGCs (Figure 2A).

By day 60, GFP immunofluorescence, colocalizing with PAX6 and HUC/D, had spread throughout the inner layer of the entire retinal organoid derived from BRN3B-GFP hiPSCs; meanwhile, WT retinal organoids displayed no GFP immunofluorescence (Figure 2B). Most cells in the outer layer of retinal organoids expressed PAX6 and SOX2, indicative of mitotic retinal progenitor cells (Figure 2B). OTX2, a marker for photoreceptor precursors, exhibited no colocalization with GFP in BRN3B-GFP organoids (Figure 2B). Overall, the BRN3B-GFP retinal organoids exhibited a layered structure and marker expression pattern similar to those of the control WT organoids. Moreover, GFP showed specific expression in RGC precursors and differentiating RGCs within the inner layer of BRN3B-GFP retinal organoids.

It has been shown previously that murine RGCs isolated from younger animals (embryonic and early postnatal stages) exhibit a better survivability post-transplantation, indicating greater plasticity and suitability for transplantation [30]. Hence, we enriched RGC precursors from 40-day BRN3B-GFP retinal organoids (Figure 2D), as GFP fluorescence emerged a few days prior, and the quantity of RGC precursors at this stage was sufficient to allow for enrichment and transplantation. At this early stage, essentially all BRN3B-expressing RGC precursors were seen to be marked by GFP (Figure 2C). We collected 40-day BRN3B-GFP retinal organoids, dissociated them into single cells, and enriched GFP^+^ RGC precursors through fluorescence-activated cell sorting (FACS) (Figure 2D,E).

### 2.3. Survival, Integration, and Differentiation of Transplanted Human RGC Precursors in Mouse Retinas

**Figure 3:**
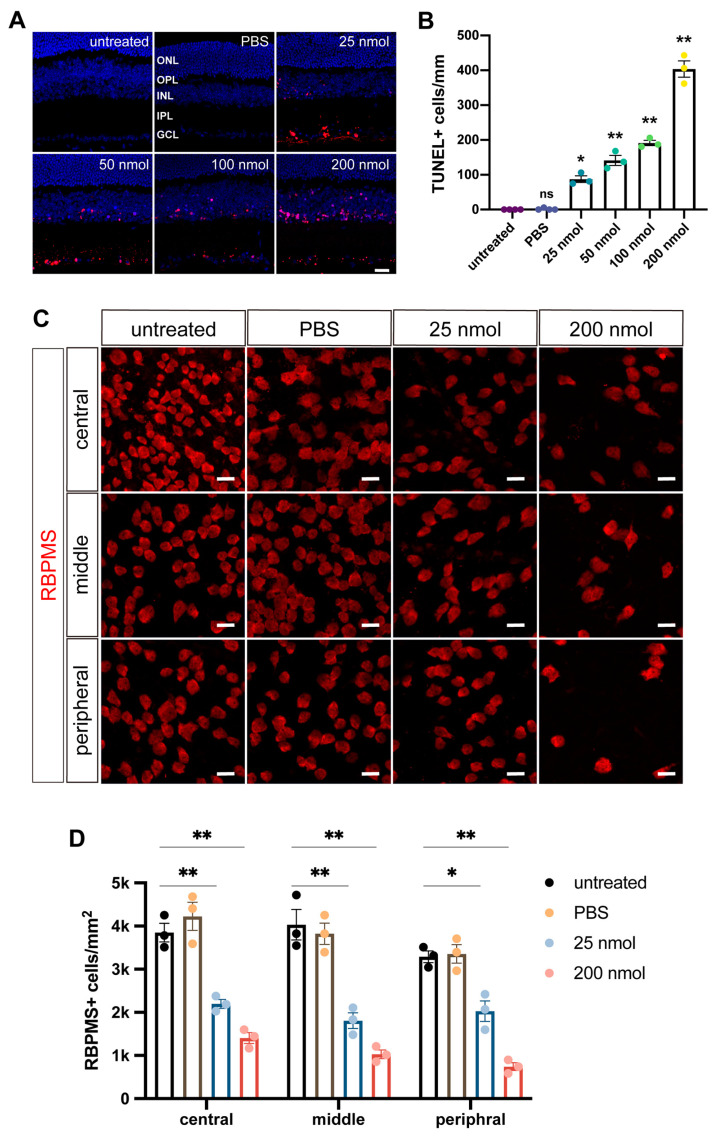
Evaluation of retinal damage post intravitreal injection of different dosages of NMDA. (**A**) TUNEL analysis of mouse retinal cryosections was performed 24 h post NMDA injection. Cells labeled with tetramethylrhodamine-deoxyuridine triphosphate (TMR dUTP, red) indicate DNA fragmentation, which signifies apoptosis. “Untreated” denotes the control group, “PBS” represents PBS injection, while 25 nmol, 50 nmol, 100 nmol, and 200 nmol refer to NMDA injection doses. The retinal layers are labeled as follows: ONL (outer nuclear layer), OPL (outer plexiform layer), INL (inner nuclear layer), IPL (inner plexiform layer), and GCL (ganglion cell layer). (**B**) Histogram showing the number of TUNEL-positive apoptotic cells in the retina post-intravitreal injection of varying doses of NMDA. Data are shown as mean ± SEM (n = 3 or 4). Statistical significance was determined using one-way ANOVA followed by Dunnett’s post hoc test: ns, not significant; * *p* < 0.001; ** *p* < 0.0001. (**C**) Representative images of RBPMS-positive RGCs in the central, middle, and peripheral regions of the retina 7 days after intravitreal injection of the indicated doses of NMDA. (**D**) Quantification of RBPMS^+^ RGCs in each indicated group in the central, middle, and peripheral regions of the retina. Data are presented as mean ± SEM (n = 3). Statistical significance was determined using two-way ANOVA followed by Dunnett’s post hoc test: * *p* < 0.001; ** *p* < 0.0001. Scale bar: (**A**,**C**) 20 μm.

N-methyl-D-aspartate (NMDA)-mediated excitotoxicity is a recognized pathway to retinal neurodegeneration, including glaucoma [31], and rapid retinal degeneration can be induced by intravitreal injection of NMDA. We injected different doses of NMDA (25, 50, 100, and 200 nmol) into the eyes of 3–4-week-old mice to determine the appropriate dose to induce glaucoma-like RGC injury. Subsequently, we performed the TUNEL assay on retinal sections 24 h post-injection to detect apoptotic cells. A dose-dependent effect of NMDA on retinal cell apoptosis was revealed by this assay (Figure 3A,B). Notably, intravitreal injection of NMDA at all tested doses led to extensive degeneration of retinal cells. The number of TUNEL-positive signals was markedly higher in the 200 nmol NMDA group, with a value over 4-fold greater than that in the 25 nmol group, and the 50 and 100 nmol groups causing intermediate numbers of apoptotic cells (Figure 3B). Moreover, 25 nmol NMDA induced apoptosis primarily in the ganglion cell layer, whereas 200 nmol NMDA also resulted in prominent cell death within the inner nuclear layer of the retina (Figure 3A).

To further validate these results, whole-mount retinas were harvested 7 days post-NMDA injection; subdivided into central, intermediate, and peripheral regions; and immunostained for the RGC-specific protein marker RBPMS (Figure 3C). Statistical analysis of RBPMS^+^ cells in the three areas revealed that the number of RGCs in the 25 nmol NMDA group was approximately halved compared to those of the untreated and PBS groups, with the greatest reduction observed in the 200 nmol NMDA group (Figure 3D). Considering that 25 nmol NMDA is sufficient to induce damage to a significant number of RGCs with minimal injury to other cell types and that excessive NMDA doses may harm transplanted RGCs, we opted for intravitreal injection of 25 nmol NMDA to make mouse models with glaucoma-like RGC injury.

**Figure 4:**
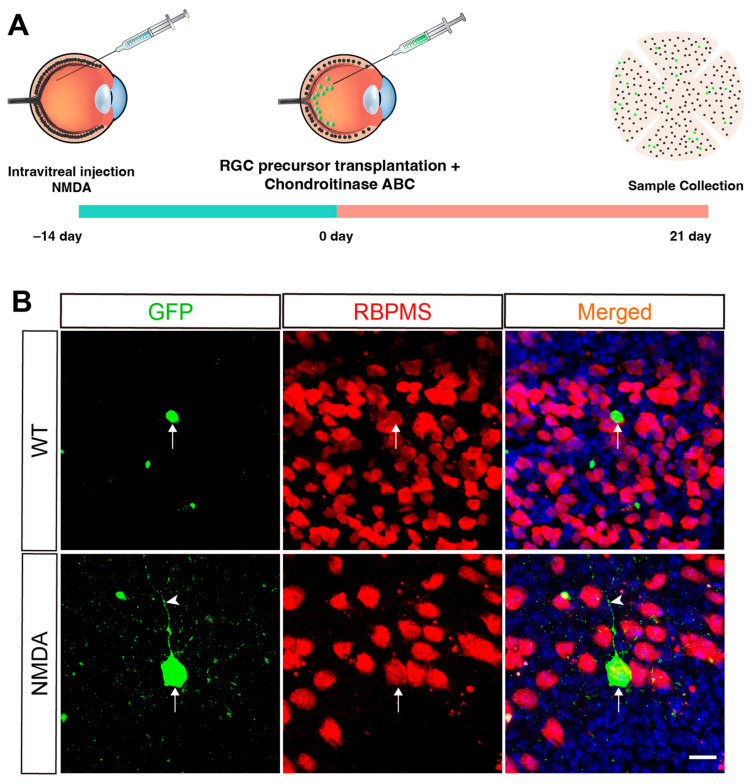
Transplantation of RGC precursors from the BRN3B-GFP retinal organoids into the mouse retina. The green dots represent GFP^+^ cells. (**A**) Timeline of NMDA injection and RGC precursor transplantation. (**B**) Transplanted whole-mount retinas from wild-type (WT) and NMDA-injured mice were double-immunostained with antibodies against GFP and RBPMS and counterstained with DAPI. Arrows point to the transplanted cells co-expressing GFP and RBPMS, and the arrowhead indicates a GFP-positive neurite. Scale bar: (**B**) 20 μm.

**Figure 5:**
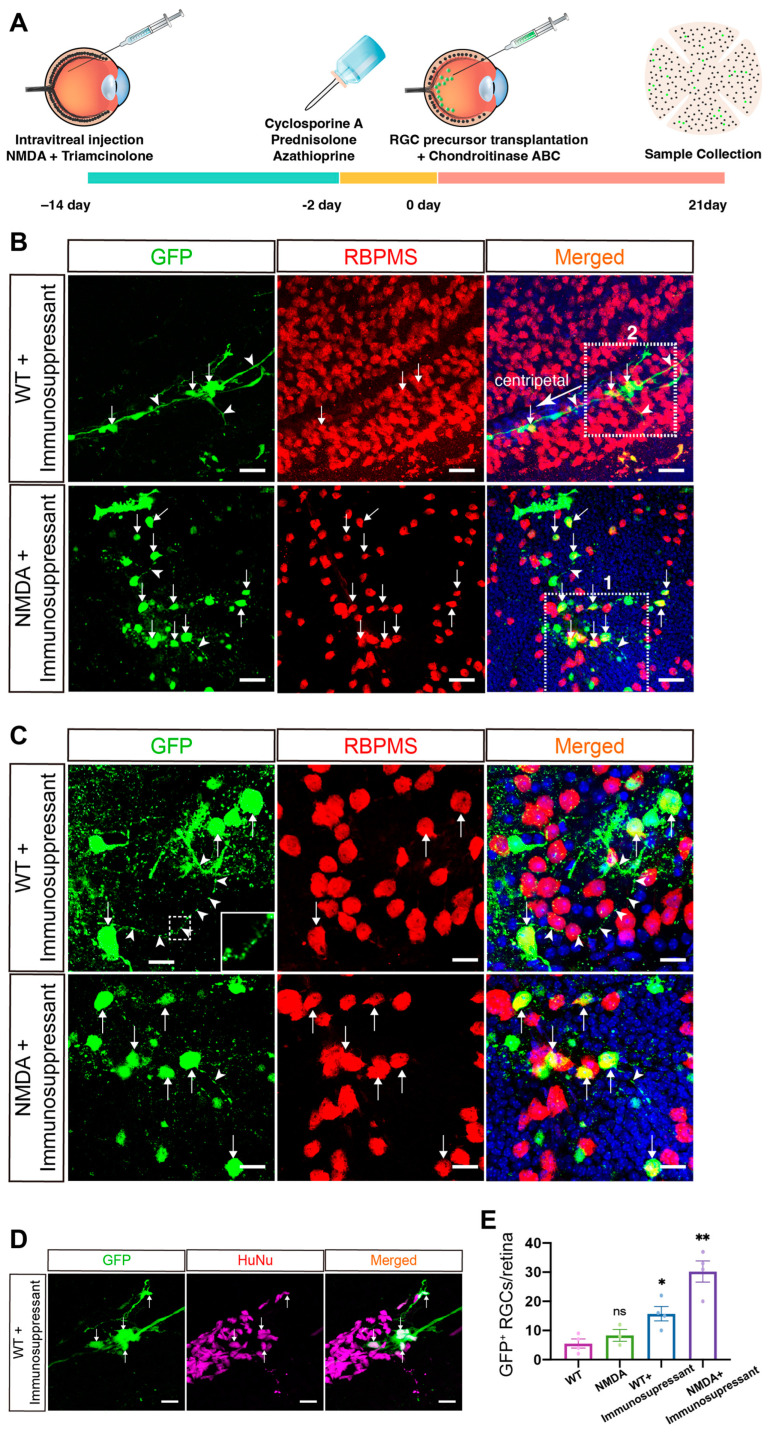
Transplantation of RGC precursors into the retinas of mice administered immunosuppressants. (**A**) Timeline of NMDA injection, immunosuppressant administration, and RGC precursor transplantation. The green dots represent GFP^+^ cells. (**B**,**C**) Transplanted whole-mount retinas from wild-type (WT) and NMDA-injured mice were double-immunostained with antibodies against GFP and RBPMS and counterstained with DAPI. The small arrows point to the transplanted cells co-expressing GFP and RBPMS, and the arrowheads indicate GFP-positive neurites/nerve bundles. The big arrow in (**B**) points to the centripetal direction. The inset in (**C**) shows a corresponding outlined region at a higher magnification to visualize an axon segment with varicosities. The lower panels in (**C**) are higher-magnification views of the outlined area 1 in (**B**). (**D**) Higher magnification views of the outlined area 2 in (**B**) show cells immunoreactive for GFP, HuNu, or both, with DAPI counterstaining. Arrows point to the transplanted cells co-expressing GFP and HuNu. (**E**) Quantification of RBPMS^+^ RGCs derived from transplanted GFP^+^ precursor cells in each indicated group. Data are presented as mean ± SEM (n = 3–4). Statistical significance was determined using one-way ANOVA followed by Dunnett’s post hoc test: ns, not significant; * *p* < 0.05; ** *p* = 0.01. Scale bar: (**C**,**D**) 20 μm; (**B**) 40 μm.

Two weeks after NMDA injection, we transplanted enriched BRN3B-GFP RGC precursors (100,000 cells per eye) into the eye’s vitreous cavity of both NMDA-injured and healthy mice (Figure 4A). Additionally, chondroitinase ABC, which induces local degradation of the extracellular matrix [14], was co-injected to promote the survival and migration of the grafted cells (Figure 4A). Three weeks post-transplantation, retinas were collected for whole-mount immunofluorescence staining for RBPMS to assess the transplantation outcome. Our results showed that the donor cells maintained GFP reporter expression which could be used to easily distinguish them from the host RGCs (Figure 4B). The success rate of transplantation was 100% in both NMDA-injured and healthy animals, with all injected retinas showing surviving GFP^+^ cells in the retinal ganglion cell layer three weeks post-transplantation, without any observed abnormal growth (Figure 4B). Some of the grafted GFP^+^ RGC precursors differentiated into more mature RGCs immunoreactive for the mature RGC marker RBPMS; moreover, some of them sprouted neurites (Figure 4B). However, in NMDA-damaged retinas, there were slightly more GFP^+^/RBPMS^+^ double-positive RGCs compared to those in WT retinas, although the difference was not significant (Figure 5E). Together, these results suggest that human RGC precursors derived from retinal organoids have the capacity to survive, migrate, and integrate into the appropriate retinal layer (ganglion cell layer) and differentiate into more mature RGCs when intravitreally transplanted into adult mice.

### 2.4. Enhancement of the Survival and Integration of Transplanted RGC Precursors with Immunosuppressants

Given the demonstrated detrimental effects of host immune response on grafted donor cells in the retina [32], we adapted the transplantation protocol to mitigate the impact of the host immune response on the survival and integration of transplanted cells. Starting 2 days before transplantation and continuing until the conclusion of the experiment, we orally administered immunosuppressants (cyclosporine A, prednisolone and azathioprine) to both WT and NMDA-injured mice (Figure 5A). Additionally, intravitreal administration of triamcinolone was performed 2 weeks prior to transplantation [13,14] (Figure 5A). For transplantation, we simultaneously injected enriched GFP^+^ RGC precursors and chondroitinase ABC into the vitreous cavity of the eye of both NMDA-damaged and healthy mice.

Three weeks post-transplantation, we collected and flat-mounted the grafted retinas for immunofluorescence staining, which revealed the presence of, apparently, more surviving GFP^+^ cells in the retinas of both WT and NMDA-injured mice compared to those without immunosuppressant treatment (Figure 4B and Figure 5B,C). Many of the grafted GFP^+^ cells expressed RBPMS, indicative of their differentiated state (Figure 5B,C). In agreement, some GFP^+^ cells extended neurites of various lengths and, occasionally, were seen to form interconnected RGC clusters that projected long axons which were fasciculated into nerve fiber bundles running in parallel to endogenous RGC nerve fibers (Figure 5B). All GFP^+^ cells could be immunolabeled by an antibody specifically against human nuclei (HuNu), confirming their human origin (Figure 5D). Notably, we observed nearly twice the number of GFP^+^ RGCs in NMDA-injured retinas compared to those in WT retinas (Figure 5E). Overall, the transplanted RGC precursors appeared to survive and integrate better in mice that received immunosuppressive therapy, and the number of differentiated RGCs present in NMDA-injured retinas nearly doubled compared to that in WT retinas, indicating that immunosuppression protects transplanted RGC precursors and that injured retinas provide a more conducive environment for the survival of the grafted cells.

## 3. Discussion

In this study, we provide preliminary evidence for the potential use of in vitro-differentiated human RGC precursors, derived from iPSCs, as a means of replacing RGCs in vivo. Our study demonstrates the survival, integration, and differentiation of hiPSC-derived donor RGC precursors post-transplantation in both healthy and damaged adult mouse retinas. Previously, similar transplantation studies have utilized primary RGCs, ESC-derived neural progenitor cells, Müller glia-derived RGC precursors, or RGCs directly induced from iPSCs, ESCs, or fibroblasts [4,13,15,30,33,34]. Our study reports the specific expression of GFP in RGC precursors of the BRN3B-GFP retinal organoids, allowing for subsequent cell enrichment without additional staining, thereby minimizing cellular damage. Moreover, compared to direct induction of RGCs in 2D culture, the steps of retinal organoid induction in our approach provide a more suitable and enriched microenvironment for RGC differentiation, mimicking the developmental process of RGCs in vivo. We opted for RGC precursors rather than mature neurons for transplantation due to the diminished potential of mature neurons in axonal outgrowth and synapse formation, which may impede their integration into the host retina post-transplantation. Consequently, RGC precursors, although functionally immature at the time of isolation, may possess a greater capacity for integration and maturation post-transplantation, thereby allowing for extensive differentiation to occur in vivo. Indeed, we found that the grafted human RGC precursors were able to survive, migrate, and integrate into the ganglion cell layer, differentiate into RBPMS^+^ RGCs, and even extend long neurites.

In this work, we observed enhanced survival of transplanted RGC precursors in NMDA-damaged mouse models, possibly due to injury-induced cues and altered local milieu that are more conducive for their survival and integration. To improve the survival and migration of transplanted cells, we considered other impeding factors as well. Previous research has highlighted inflammatory microglia and chondroitin sulfate proteoglycans as natural barriers to cell survival and migration into degenerated retinas [14]. Given the xenogeneic nature of the human RGC precursors, we supplemented our transplantation procedure with oral immunosuppression, as well as local anti-inflammatory and extracellular matrix degradation therapies, to more effectively overcome these barriers [14]. With these treatments, GFP^+^ cells were seen within the ganglion cell layer that expressed RGC-specific markers and grew neurites following transplantation, suggesting successful migration and integration of human RGC precursors into the murine host retina.

In recent years, a significant concern has emerged regarding cell fusion and material transfer observed in photoreceptor replacement studies, which has prompted researchers to question the validity of numerous prior studies [35,36,37,38]. In our investigation, the use of a specific human nucleus stain to distinguish grafted donor cells from host cells effectively refutes cellular fusion as a viable explanation. Furthermore, examination of transplanted cells integrated within the ganglion cell layer revealed the presence of only single nuclei, as confirmed by 4′,6-diamidino-2-phenylindole (DAPI) staining. Therefore, unlike photoreceptor precursors, human RGC precursors from the retinal organoids display no detectable cell fusion, suggesting that material transfer may be a phenomenon associated with specific cell types.

While our study showed that transplanted RGC precursors had the ability to integrate and differentiate in the host retina, it remains unclear whether the differentiated RGCs are functional. By patch clamp recording and other techniques, future investigations may explore the electrophysiological properties of transplanted cells and assess their connectivity with other retinal neurons. Additionally, comparing visual evoked potentials (VEPs) between mice with and without transplanted cells post-NMDA injury may be able to determine if transplanted RGC precursors enhance VEP. Undoubtedly, our study provides an initial exploration for future cell transplantation therapy using hiPSC-derived RGC precursors, offering valuable insights into the feasibility of cell replacement therapy for degenerative retinal diseases involving RGC loss.

In addition to cell transplantation, in vivo neuronal regeneration through reprogramming presents another viable approach toward treating retinal degeneration. Neurons regenerated through this method may have the advantage of easier migration to correct locations and more accurate projection to intended targets. Recent studies have shown significant progress in this area. For instance, RGCs were reprogrammed from Müller glial cells by overexpressing relevant developmental regulators Ascl1, Brn3b, and Isl1 [39,40]. Thus, despite numerous obstacles and unresolved issues in treating degenerative retinal diseases, both in vitro and in vivo neuronal regeneration approaches may have the potential to replace damaged retinal cells. In this work, the establishment of the BRN3B-GFP hiPSC line and cultivating its derived retinal organoids have simplified the process of obtaining enriched RGC precursors. And transplantation of reporter-marked RGC precursors offers an appealing and practical approach for basic and translational studies of RGC degeneration associated with various retinal diseases such as glaucoma and other optic neuropathy.

## 4. Materials and Methods

### 4.1. Maintenance of hiPSCs

When hiPSC clones reached approximately 80% confluency, they were dissociated using 50 mM EDTA for 5 min at 37 °C. Subsequently, the dissociated cells were plated at a dilution of 1:10 into a fresh 1% Matrigel (Corning, New York, NY, USA)-coated 6-well plate and then cultured in mTeSR1 medium (Stem Cell Technology, Vancouver, BC, Canada). The medium was refreshed daily with mTeSR1 medium, and iPSCs were passaged every 4–5 days depending on confluency.

### 4.2. Generation of BRN3B-GFP hiPSC Line

The BRN3B-GFP hiPSC cell line was generated from the UiPSC-001 hiPSCs using CRISPR/Cas9 gene editing as described previously [19,20] (Figure 1A). In brief, sgRNAs were constructed into the pX459 plasmid (Addgene: #62988, Watertown, MA, USA). The homologous recombination arms were amplified from the genomic DNA of normal iPSCs and subsequently inserted into the pBluescript II SK (+) vector. Within the template plasmid, the termination codon of *BRN3B* was replaced with the P2A-eGFP fragment. Following this, the sgRNA and template plasmids were introduced into the UiPSC-001 hiPSCs by electroporation. The cells were then cultured in mTeSR1 medium supplemented with 300 ng/mL puromycin for 48 h, with daily medium changes. After puromycin selection, individual monoclonal colonies were isolated and expanded in wells of a 12-well plate containing mTeSR1 medium. Genomic DNA was extracted from a subset of cells at 80% confluence, and PCR was performed to determine the genotype, followed by verification through DNA sequencing.

### 4.3. Karyotyping of hiPSCs

G-banded karyotype analysis was conducted following established procedures [41]. The BRN3B-GFP hiPSCs were cultured in mTeSR1 until reaching 80–90% confluency. The cells were then treated with colchicine (0.2 μg/mL) in mTeSR1 for 2 h, enzymatically lifted with Accumax (Millipore, Billerica, MA, USA), and centrifuged after washing with DPBS (Gibco, New York, NY, USA). Following resuspension in hypotonic solution (0.075 M KCl) and incubation, the cells were fixed with methanol–acetic acid (3:1), dropped onto wet slides, and GTG-banded for analysis.

### 4.4. Induction of 3D Retinal Organoids from hiPSCs

Retinal organoids were generated from hiPSCs using a previously established protocol [29]. In short, hiPSCs at 80% confluency were dissociated into single cells with the TrypLE Express enzyme (Gibco, New York, NY, USA) and reaggregated in low-adhesion 96-well plates with V-bottom conical wells (Sumilon PrimeSurface plate MS-9096VZ; Sumitomo Bakelite, Tokyo, Japan) in Medium 1 (14,000 cells per well, 100 μL) supplemented with 2 μM Thiazovivin (TOCRIS, Bristol, UK). Medium 1 contains Iscove’s modified Dulbecco’s medium (IMDM, Gibco, NY, USA) and Hams F12 (F12, Gibco, NY, USA) in a 1:1 volume ratio, 10% knockout serum replacement (KSR) (Gibco, NY, USA), 1% Glutamax (Gibco, NY, USA), 1% chemically defined lipid concentrate (Gibco, NY, USA), 450 μM monothioglycerol (Sigma-Aldrich, St. Louis, MO, USA), and 1% penicillin and streptomycin (Gibco, NY, USA). On day 3, medium 1 was replenished with 50 μL per well. Recombinant human BMP4 (R&D Systems, NE Minneapolis, MN, USA) was added on day 6 to a final concentration of 1.5 nM (55 ng/mL), followed by subsequent dilutions with half-medium exchanges every three days until day 18.

On day 18, neural retina (NR)-like spheroids were transferred to an ultra-low attachment dish (Sumilon PrimeSurface dishes MS-90900Z, Sumitomo Bakelite, Japan) and cultured in Medium 2 for retinal pigment epithelium induction. Medium 2 contains DMEM/F12-Glutamax medium (Gibco, NY, USA), 1% N-2 supplement (Gibco, NY, USA), 1% penicillin and streptomycin, 3 μM CHIR99021 (Selleck, Houston, TX, USA), and 5 μM SU5402 (Selleck, Houston, TX, USA). On day 24, NR-like tissues were fragmented using ophthalmic forceps and scissors under an inverted microscope (Leica, Wetzlar, Germany) and transferred to a 6-well ultra-low attachment plate (Corning, NY, USA) for further culture in Medium 3 for maturation of retinal organoids, with regular medium changes every three days. Medium 3 contains DMEM/F12-Glutamax medium, 1% N-2 supplement, 10% fetal bovine serum (FBS) (Genial, Brighton, CO, USA), 0.5 mM retinoic acid (Sigma-Aldrich, St. Louis, MO, USA), 0.1 mM taurine (Sigma-Aldrich, St. Louis, MO, USA), and 1% penicillin and streptomycin.

### 4.5. Dissociation of Retinal Organoid Cells and Enrichment of RGC Precursors

On day 40, the BRN3B-GFP retinal organoids (~120–150 organoids) were collected into a 15 mL conical tube. Following the addition of 5 mL Accumax with 1 mg/mL DNase I (Roche, Basel, Switzerland), the organoids were incubated at 37 °C for 1 h. Subsequently, gentle pipetting with a 1 mL pipette dissociated the organoids into single cells. After the addition of 10 mL DPBS with 10% FBS, the cell suspension was filtered through a 40 μm cell strainer into a 15 mL conical tube. The cells were precipitated by centrifugation at 500× *g* for 5 min, followed by two washes with DPBS. They were suspended in 500 μL of DPBS containing 2% FBS and 1 mM EDTA and sorted using the FACSAria Fusion cell sorter (BD Biosciences, Franklin Lake, NJ, USA) via fluorescence-activated cell sorting (FACS).

### 4.6. Transplantation of RGC Precursors

All mouse experiments adhered to the IACUC standards and were approved by Zhongshan Ophthalmic Center of Sun Yat-sen University. C57BL/6 mice were obtained from the Shanghai Model Organisms Center, Inc. Mice of 3–4 weeks of age mice were used for transplantation. For the NMDA injury group, mice received an intravitreal injection of 25 nmol of NMDA two weeks prior to transplantation. Additionally, for the immunosuppressive treatment group, mice received intravitreal injection of 160 μg triamcinolone (TA), or TA combined with 25 nmol NMDA (Abcam, Cambridge, UK). These mice were also immunosuppressed with oral cyclosporine A (210 mg/L), prednisolone (5 mg/L), and azathioprine (20 mg/L); these were administered daily in drinking water starting two days before transplantation and continued until the experimental endpoint, as described previously [13,42]. For cell transplantation, mice were anesthetized via intraperitoneal injection of 1% pentobarbital sodium (~60 mg/kg), followed by dilation and surface anesthesia of the eyes. A total of 1 × 10^5^ RGC precursors, obtained through FACS, combined with 0.01 unit of chondroitinase ABC (Sigma-Aldrich, St. Louis, MO, USA), were injected into the vitreous cavity of each eye under a surgical microscope. After injection, eyes were treated with erythromycin eye ointment to prevent infection, and mice were placed on a warming pad until recovery and then returned to their housing cages. At the experimental endpoint, 3 weeks post-transplantation, animals were euthanized by CO_2_ inhalation followed by cervical dislocation. Eyes were enucleated, fixed in 4% paraformaldehyde for 1 h, and processed for retinal whole-mount preparation.

### 4.7. Immunohistochemistry

Immunostaining of retinal organoid sections and whole-mount retinas was performed as previously described [43,44]. In brief, mouse eyeballs were fixed in 4% paraformaldehyde (PFA) (Sigma-Aldrich, St. Louis, MO, USA) for 1 h at 4 °C. The retinas were carefully dissected under an inverted microscope. The retinas were processed directly for staining and subsequent experiments. After three washes with PBST (PBS with 0.25% Triton X-100) (Sigma-Aldrich, St. Louis, MO, USA), samples were blocked in PBST containing 5% donkey serum for 1 h. Primary antibodies, diluted in the blocking solution, were applied, and the samples were incubated at 4 °C for 48 h. Following three additional PBST washes, retinas were incubated with diluted secondary antibodies and 4′,6-diamidino-2-phenylindole (DAPI) for 2 h in the dark. After a final series of washes, retinas were cut into a cloverleaf shape (four quadrants), mounted with mounting medium (Polyscience, Warrington, UK), and carefully covered with a coverslip.

The procedure for handling organoids involved their spontaneous aggregation in 1.5 mL Eppendorf tubes. Subsequently, the organoids were washed twice with DPBS. Fixation was performed using 4% PFA for 15 min at room temperature. Following fixation, the organoids were washed three times with DPBS. They were then dehydrated in 30% sucrose solution (Sigma-Aldrich, St. Louis, MO, USA) overnight at 4 °C. After dehydration, the organoids were embedded in tissue freezing medium (Leica, Wetzlar, Germany) and sectioned at 14 μm using a cryostat microtome. The sections were allowed to air-dry at room temperature overnight. Immunostaining of organoid sections was carried out as described above, with the exception that the primary antibody incubation time was shortened to 24 h at 4 °C. Primary antibodies included goat anti-GFP (Abcam, Cambridge, UK, ab6673, 1:1000), rabbit anti-PAX6 (Cell Signaling Technology, Danvers, MA, USA, 60433, 1:500), goat anti-BRN3B (Santa Cruz Biotechnology, Dallas, TX, USA, sc-6026, 1:100), mouse anti-HUC/D (Invitrogen, Carlsbad, CA, USA, A-21271, 1:500), goat anti-OTX2 (R&D Systems, NE Minneapolis, MN, USA AF1979, 1:1000), rabbit anti-SOX2 (Cell Signaling Technology, Danvers, MA, USA, 3579, 1:1000), rabbit anti-RBPMS (Novus, Littleton, Colorado, USA, NBP2-20112, 1:500), and mouse anti-nuclei (HuNu) (Millipore, Billerica, MA, USA, MAB1281, 1:1000). Secondary antibodies included donkey anti-rabbit, donkey anti-goat, and donkey anti-mouse Alexa488 IgG and Alexa594 IgG (1:1000; Invitrogen, Carlsbad, CA, USA). Nuclear counterstaining was performed using DAPI (Invitrogen). Imaging was conducted using a laser scanning confocal microscope (Carl Zeiss, Oberkochen, Germany, LSM700).

### 4.8. Quantification and Statistical Analysis

Retinal samples from a minimum of three mice per group were analyzed (n ≥ 3). For the quantification of TUNEL^+^ cells or RBPMS^+^ cells, 6–8 non-overlapping optic fields were selected from similar regions of each retinal section or flat-mount. Optic fields were defined as the area captured using laser confocal microscopy at consistent magnification, and the counts from these fields of each retina were averaged to represent a single data point. For GFP^+^ cells, the number of all GFP^+^ cells in each whole-mount retinal sample was counted under confocal microscopy to constitute a single data point. Statistical analyses were conducted using GraphPad Prism 8.0 (GraphPad Software, San Diego, CA, USA), and data are presented as mean ± SEM. The following statistical tests were applied: for Figure 3B and Figure 5E, a one-way ANOVA was conducted followed by Dunnett’s post hoc test. For Figure 3D, a two-way ANOVA was performed followed by Dunnett’s post hoc test.

## Data Availability

The data presented in this study are available in the article.
